# Red blood cell aggregates and their effect on non-Newtonian blood viscosity at low hematocrit in a two-fluid low shear rate microfluidic system

**DOI:** 10.1371/journal.pone.0199911

**Published:** 2018-07-19

**Authors:** Rym Mehri, Catherine Mavriplis, Marianne Fenech

**Affiliations:** Department of Mechanical Engineering, University of Ottawa, Ottawa, Ontario, Canada; Université Claude Bernard Lyon 1, FRANCE

## Abstract

Red blood cells (RBCs) are the most abundant cells in human blood. Remarkably RBCs deform and bridge together to form aggregates under very low shear rates. The theory and mechanics behind aggregation are, however, not yet completely understood. The main objective of this work is to quantify and characterize RBC aggregates in order to enhance the current understanding of the non-Newtonian behaviour of blood in microcirculation. Suspensions of human blood were flowed and observed *in vitro* in poly-di-methyl-siloxane (PDMS) microchannels to characterize RBC aggregates. These microchannels were fabricated using standard photolithography methods. Experiments were performed using a micro particle image velocimetry (μPIV) system for shear rate measurements, coupled with a high-speed camera for flow visualization. RBC aggregate sizes were quantified in controlled and measurable shear rate environments for 5, 10 and 15% hematocrit. Aggregate sizes were determined using image processing techniques, while apparent viscosity was measured using optical viscometry. For the samples suspended at 5% H, aggregate size was not strongly correlated with shear rate. For the 10% H suspensions, in contrast, lowering the shear rate below 10 s^-1^ resulted in a significant increase of RBC aggregate sizes. The viscosity was found to increase with decreasing shear rate and increasing hematocrit, exemplifying the established non-Newtonian shear-thinning behaviour of blood. Increase in aggregation size did not translate into a linear increase of the blood viscosity. Temperature was shown to affect blood viscosity as expected, however, no correlation for aggregate size with temperature was observed. Non-Newtonian parameters associated with power law and Carreau models were determined by fitting the experimental data and can be used towards the simple modeling of blood’s non-Newtonian behaviour in microcirculation. This work establishes a relationship between RBC aggregate sizes and corresponding shear rates and one between RBC aggregate sizes and apparent blood viscosity at body and room temperatures, in a microfluidic environment for low hematocrit. Effects of hematocrit, shear rate, viscosity and temperature on RBC aggregate sizes have been quantified.

## Introduction

Red blood cells (RBCs) play a key role in dictating the rheological behaviour of blood in microcirculation. They are responsible for the non-Newtonian behaviour of blood, due to their unique properties; solid but pliable RBCs aggregate and form three-dimensional cell clusters and one-dimensional “rouleaux” stacks that account for the variation of blood viscosity in microcirculation, as they flow, tumble, form, disband and squeeze through small blood vessels. Factors affecting rouleaux formation in turn affect blood viscosity. These factors include the composition of the suspension medium, the elasticity of RBC membranes, the hematocrit of blood and the applied shear rate. *In vivo*, blood viscosity is further affected by the geometry and diameter of the vessels; the apparent viscosity decreases with decreasing vessel diameter in a phenomenon called the Fåhraeus-Lindqvist effect [1]. A further consequence of this effect is that blood in microcirculation also exhibits a decrease of local hematocrit with decreasing vessel diameter [2]. This is due to plasma skimming effects in microcirculation caused by the formation of the cell free layer, which greatly influences the Fåhraeus-Lindqvist effect [3]. In fact, it has been shown that the local hematocrit, defined as the percentage by volume of RBCs (denoted H), in microcirculation does not exceed 20% H, compared to 45% H typical of macrocirculation [4].

Early experimental studies measured blood apparent viscosity using Couette or coaxial viscometers, for varying shear rates [5–7], including for very low shear rates near and at zero [8], varying aggregation levels (by varying fibrinogen concentration) [5], and varying hematocrit [6]. They found that, in general, blood apparent viscosity increases with increased aggregation [5] as well as with increased hematocrit [6]. Merrill [5] showed that the yield stress of blood is strongly affected by the fibrinogen content in the blood as well as the proteins in the plasma.

### Previous non-Newtonian models

Several non-Newtonian models have been developed to describe the measured viscous behaviour of blood. One of the most known and used non-Newtonian models for blood is the power law or Ostwald-de Waele power law [9]. According to this model, the fluid shear stress, *τ*, can be calculated as:

$$\tau =K{\left(\frac{du}{dy}\right)}^{n}$$ (1)

where *K* represents the fluid consistency index, *n* the non-Newtonian behaviour index, *u* the streamwise velocity of the fluid and *y* the coordinate normal to the vessel wall. This model, however, does not accurately predict the behaviour and viscosity of fluids at very high or very low shear rates: at these extremes, the viscosity, estimated by the power law model, tends to infinity rather than reaching a constant value as observed experimentally [10]. Despite this limitation, the power law model is still widely used for its simplicity.

The Casson model [11] characterizes a Non-Newtonian fluid exhibiting both shear-thinning behaviour and a yield stress, simultaneously. It models the shear stress as:

$$\begin{array}{c} \sqrt{\tau }=\sqrt{\tau_{y}}+\sqrt{k\frac{du}{dy}}\;\text{if}\;\tau >\tau_{y},\\ \frac{du}{dy}=0\;\text{if}\;\tau \leq \tau_{y}, \end{array}$$ (2)

where *k* is the Casson model constant and *τ*_*y*_ is the yield stress of blood. Cokelet [8] found that the Casson model describes blood viscosity well at very low shear rates, except that it does not account for the effects of hematocrit (e.g. the influence of hematocrit on the yield stress) nor the effects of inter-aggregate forces.

The Carreau-Yasuda model, first introduced by Pierre Carreau [12] and further developed by Kenji Yasuda [13], models the apparent viscosity of the non-Newtonian fluid as:

$$\mu_{app}=\mu_{\infty }+(\mu_{0}-\mu_{\infty }){\left(1+{\left(\lambda \dot{\gamma }\right)}^{a}\right)}^{\frac{n-1}{a}},$$ (3)

where $\dot{\gamma }$ represents the shear rate, *μ*_*∞*_ the viscosity at infinite shear, *μ*_*0*_ the viscosity at zero shear, *λ* the relaxation time, *n* the power law index and *a* the shape parameter. The Carreau model represents a special case of the Carreau-Yasuda model in which *a* = 2. At high shear rates, the fluid thus behaves as a Newtonian fluid with a viscosity of *μ*_*∞*_, whereas at low shear rates, the fluid acts as a Newtonian fluid with a viscosity of *μ*_*0*_. The Carreau-Yasuda model is capable of predicting the shear-thinning behaviour of blood, however the thixotropic (time-dependent) behaviour of blood is not captured. Several other models have attempted to mimic various aspects of blood behaviour, such as hematocrit dependence and yield stress behaviour, mostly at the macroscale [14–16]. Although it has been shown through experimental studies that these models can predict the behaviour of blood to some level of accuracy [17], none of these account for the effects of RBC aggregates, which greatly influence the micro-rheological behaviour of blood [18–20].

Bureau et al. [21] presented a dynamical study of blood to investigate its rheological behaviour in transient flow regimes at low shear rates. Several experiments were performed to determine the stress variation in time when the systems were subjected to incremental rectangular and triangular shear rates (hysteresis cycles). They discovered that, dynamically, blood exhibits a viscoelastic, shear-thinning and thixotropic behaviour, simultaneously.

Owens [22] developed a new constitutive equation describing the behaviour of whole blood based on the linear spring law and the multi-mode generalized Maxwell equation, including RBC aggregation as a parameter in the model. The model was compared to the study of Bureau et al. [21] and showed similar results when subjected to incremental triangular shear rates.

Fedosov et al. [23] developed a numerical model of blood dynamics that predicted the dependence of blood viscosity on shear rate and hematocrit (at 20%, 30% and 45% H). Their model incorporated coarse-grained molecular dynamics with RBC mechanics and simulated the formation of rouleaux. Their simulations of blood (at 45%H and 37C) for shear rates between 0.005 and 1000 s^-1^ predicted a steep increase in viscosity at very low shear rates owing to the significant formation of RBC aggregates, and closely matched the experimental results of Merrill et al. [5], Chien et al. [6], and Skalak et al. [7].

### Optical viscometry

The viscosity within *in vitro* microchannels can be measured using the optical viscometry method [24]. This technique is based on the analytical solution for two co-flowing fluids in a rectangular channel, which can be obtained by a Fourier series expansion. This method was first developed by Galambos and Forster [25] and was investigated in a transparent T-junction device to estimate the viscosity of a Newtonian fluid, based on the known viscosity of a reference fluid. The reference fluid was mixed with a fluorescent dye so that the fluid interface could be clearly detected. High flow rates were used to minimize the diffusion between the two miscible fluids. Later, Guillot et al. [26] extended the technique in a Y-shaped device to measure the apparent viscosity of non-Newtonian fluids using immiscible fluids to prevent the fluids mixing at very low shear rates. The use of immiscible fluids, however, requires detailed knowledge of the shape of the interface between the fluids, which requires a more extensive investigation. For this purpose, a fluorescent confocal microscope was used to map the three-dimensional shape of the fluid interface. Solomon and Vanapalli [27] extended the technique and designed a multiplexed viscometer allowing measurements of viscosity for up to eight samples simultaneously, using miscible Newtonian and non-Newtonian fluids.

### Objectives

Few experimental studies provide details on RBC aggregate sizes and behaviour [28–31]. Most modelers have only the results of the classical references measuring blood viscosity in macrocirculation to test their theories against, and most of these are for 45%H [5–7, 32, 33]. The present work investigates microcirculation conditions through the application of recent, affordable and simple microfabrication techniques. A two-fluid shear flow microchannel system allows for direct measurement of blood velocities and RBC aggregate sizes, under controlled shear rate conditions and at hematocrit levels close to those typical of microcirculation. We use the optical viscometry technique to measure the apparent viscosity of blood in a Y-microchannel system while taking simultaneous measurements of shear rates and aggregate sizes. This work establishes a sound methodology for experimental RBC aggregate analysis and provides new, more detailed experimental data for the blood rheology literature.

## Materials and methods

### Experimental set up

**Fig 1:**
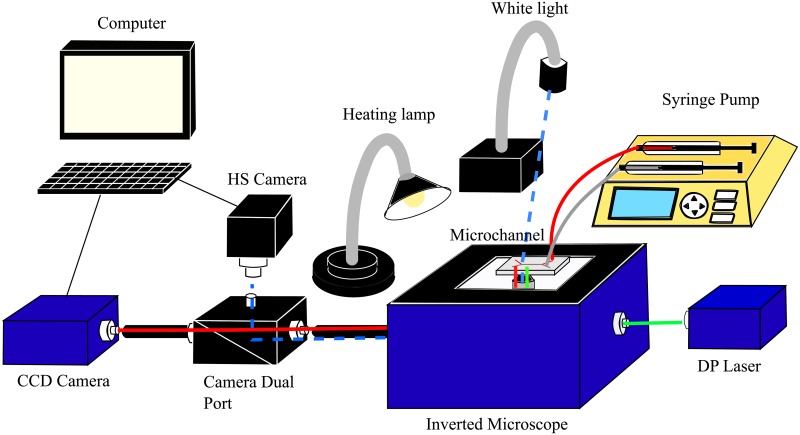
Experimental set-up and light path within the system.

The experimental set-up (Fig 1) consisted of a μPIV system (MITAS; LaVision, Germany) coupled with a near infrared high-speed camera (Basler ace, acA2000-340km-NIR; BASLER, Germany) controlled using LabVIEW software (National Instruments, USA), and a temperature control system controlled using Arduino software. The μPIV system comprised a charged-coupled device (CCD) camera (Imager Intense; LaVision), a Nd:YAG laser (Solo-II; New Wave Research, USA) emitting a wavelength λ_emission_ = 532 nm and an inverted microscope (MITAS, LaVision) with 20X lens magnification. The 20X objective lens provided a numerical aperture of 0.4 and a depth of field of 4 μm. In order to control the position of the microchannel relative to the measurement plane, the μPIV set-up included a moving stage (xyz directions) with a minimum step size of 1 μm, controlled by Davis Imaging Software (LaVision GmbH, Germany). Tracer particles were mixed with the fluid samples (diluted at 1% in water, *d*_*p*_ = 0.79 μm, *λ*_*abs*_ = 542 nm and *λ*_*emission*_ = 612 nm), which illuminated when exposed to the appropriate wavelength. Each fluid sample, drawn into a glass syringe or 100 μL) (Hamilton, USA), was injected at different flow rates into a disposable PDMS microchannel (110 μm in width and 60 μm in depth) using a pressure driven pump (Nexus3000, Chemyx, USA).

Blood flow was observed in the microchannel, normal to the velocity gradient, providing a different angle for the flow investigation than used in previous studies, such as the one by Kaliviotis and Yianneskis [34], in which aggregates were visualized in the direction of velocity variation. A dual camera port directed the light path to the different cameras: white light was directed to the high-speed camera for imaging the blood aggregates, whereas the light emitted from the laser was directed to the CCD camera for the μPIV measurements. For the μPIV measurements, 100 image pairs were recorded at 5 Hz, the results of which were averaged to obtain the average velocity field. The spatial resolution of the CCD camera was 0.27 μm/pixel when using the 20X lens. For imaging the RBC aggregates, high-speed images having resolution 0.2 μm/pixel were recorded at 160 frames/s.

Throughout the experiment, the temperature of the blood sample was monitored and controlled using a custom-made temperature control system. The system comprised a heating lamp, a dimmer circuit to modify the lamp temperature, a thermocouple (Adafruit, USA) as a feedback sensor and a microcontroller (Adafruit, USA). The system monitored and adjusted the heating lamp every 2 seconds to maintain the target temperature. To acquire an accurate temperature reading without affecting the flow, the thermocouple was positioned at the outlet port of the microchannel, ensuring no contact with the channel walls.

### Experimental procedure

**Fig 2:**
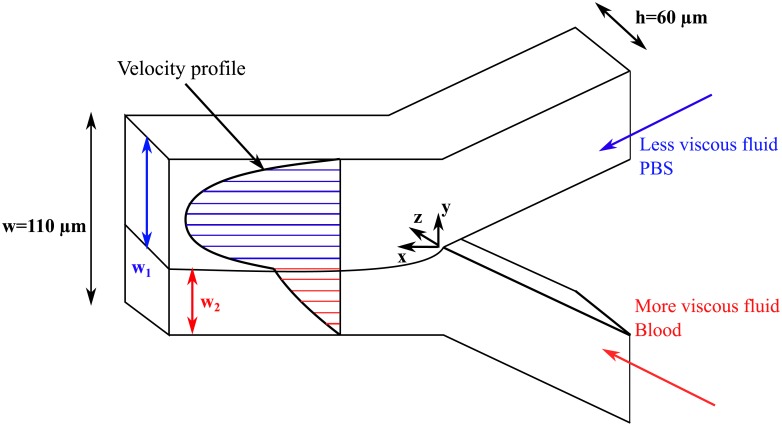
Blood entrained by phosphate buffered saline (PBS) in a double Y-microchannel configuration.

Human RBC aggregates were visualized and investigated in Y-microchannels (Fig 2). The microchannels were fabricated using standard photolithography methods [35]. The PDMS microchannel fabrication is inexpensive and allows easy bonding to different surfaces. Additional details of the fabrication process are provided in Mehri et al. [36]. Each RBC suspension was tested at five different flow rates ranging from 5 to 35 μL/hr to quantify the effect of shear rate on RBC aggregate sizes in controlled shear flows and at two different temperatures: 23°C (room temperature) and 37°C (body temperature). The flow rate range was chosen in order to provide shear rates in the blood layer within the ranges of blood aggregation (1–50 s^-1^). To shear the fluid, blood entering from the bottom branch of the microchannel was entrained by a phosphate buffered saline (PBS) solution, which entered from the top branch at a flow rate four times higher than that of the blood (Fig 2). The flow was imaged using the high-speed camera while the velocity field was measured using the μPIV system. This set-up provided measurable, constant and controlled shear rates within the range at which blood aggregates [37]. Shear rates were calculated from the slopes of the linear velocity profiles that were measured within the blood fluid layer. The blood apparent viscosity was estimated from the velocity profiles measured in the branches using the optical viscometry method mentioned in the introduction and further detailed in the viscosity measurements section. The viscosity data were fitted using two empirical models commonly used to model blood flow: the power law and Carreau models [9, 13] (also described in the introduction). These models are widely used in the literature for their simplicity [38, 39]. The high-speed images of the RBC aggregates were processed in MATLAB, using techniques described in Mehri et al. [40].

### Blood sample preparation

Human blood was collected from 11 healthy adult volunteers (4 males and 7 females) in 2015 with the approval of the University of Ottawa ethics committee (H11-13-06). Informed consent was obtained in writing from each of the participants. The blood was collected in 4 mL tubes coated with ethylenediaminetetraacetic acid (EDTA) to prevent coagulation. All samples (whole blood) were centrifuged three times, following standard procedures, each for 10 minutes at 3000 rpm in order to separate the blood constituents. Blood plasma was collected only from the first centrifugation. The plasma was then filtered using a 0.2 μm plasma filter (Nylon Non-Sterile, Fisher Scientific, Ireland) to ensure no white blood cells and platelets are present without interfering with the plasma proteins. Removing these constituents for experiments facilitates the use of μPIV, improves the image quality and prevents blood clot formation. The RBCs of each sample were then suspended in their original plasma at three hematocrits (5%, 10%, and 15%) in order to measure the effect of hematocrit on the aggregate sizes. The reported hematocrits in this study represents the sample hematocrit at the entrance of the feeding tube To verify the hematocrit of each suspension, a capillary tube filled with a sample of each suspension was centrifuged in a microcentrifuge (CritSpin, Thermo Fisher Scientific, China) to determine the volume of the separated RBCs in the tube. Red fluorescent tracer particles (diameter = 0.79 μm; Fluoro-Max, USA) were added to the RBC suspensions for the velocity measurements: 60 μL of an aqueous particle solution (1% solids) was added to each 1 mL suspension, resulting in a 0.06% particle concentration. The experiments were performed within eight hours after blood collection.

### Velocity measurement

**Fig 3:**
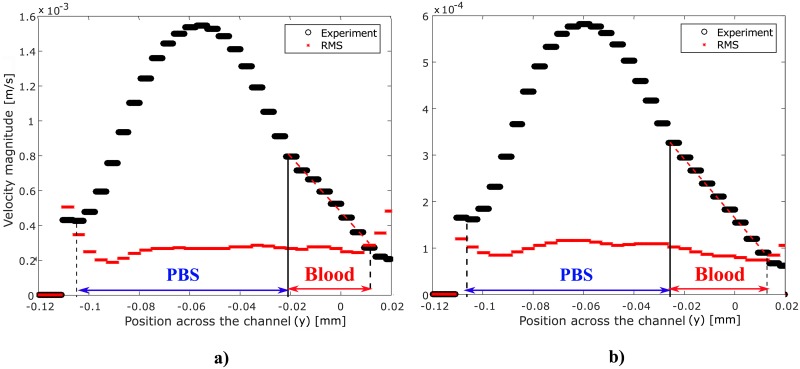
Velocity profiles (black data points) averaged temporally and spatially and extracted from the vector fields at x = 1.3 mm from the intersection of the two fluidsfor RBCs suspended at 10% H flowing with (a) Q = 20 μL/hr and (b) Q = 7.5 μL/hr. The red data points show the Root Mean Square (RMS) associated with the velocity measurements. The slope of the red dashed line indicates the shear rate within the blood layer.

Velocities inside the microchannels were measured using the μPIV system. The methodology used for the velocity field measurements was previously validated for the study of blood micro-flows in a study by Pitts et al. [41]. In that study, different μPIV pre-processing and processing methods were compared for flows in two microchannel geometries. Based on that investigation, the cross-correlation method with image background removal was selected for the present measurements. A multi-pass approach was used for the velocity calculations within the correlation window, starting from a correlation window size of 64 × 64 pixels^2^, and decreasing to a window size of 32 × 32 pixels^2^ with a 50% overlap for the different passes. The correlation windows were weighted in the *x*-direction with a ratio of 4:1 corresponding to the direction of the flow in the microchannel. The time between the laser pulses, *dt*, was varied between 2 and 15 ms depending on the flow rate in the channel (5< = *Q* < = 35 μL/hr) in order to cause particle displacements between 6 and 10 pixels between consecutive frames. The *dt* was optimized for measurements in the blood layer in order to obtain accurate shear rate measurements within the blood layer. To verify the accuracy of the velocity measurements, the root mean square (RMS) of the velocity was calculated for the 100 images of each sample as:

$$RMS=\sqrt{\frac{N\sum_{i=1}^{N}u_{i}^{2}-N\sum_{i=1}^{N}u_{i}}{N(N-1)}},$$ (4)

where *N* is the total number of images and *u*_*i*_ is the velocity value of image *i*. The results were given in terms of pixel displacement which were then scaled to the units of velocity (mm/s) based on the time interval between the images pairs, *dt*, and the scale factor *α*_*c*_ determined by the calibration of the CCD camera. The velocity profiles were averaged in time (for *N* = 100) and then averaged again along the streamwise direction, along the length of the visualization window, to produce a single representative profile for the measurement, as shown in Fig 3. The velocity profiles were measured at a distance of 1.3 mm from the intersection of the two fluids. The shear rates for each measurement were calculated as the slope of the 2D velocity profile at the channel mid-plane within the blood layer (Fig 3). The boundaries of the blood layer were identified based on the visualization of the fluorescent particles measured with the μPIV system. Further details of the methodology are provided in the study of Mehri et al. [40].

### Aggregate detection and image processing technique

**Fig 4:**
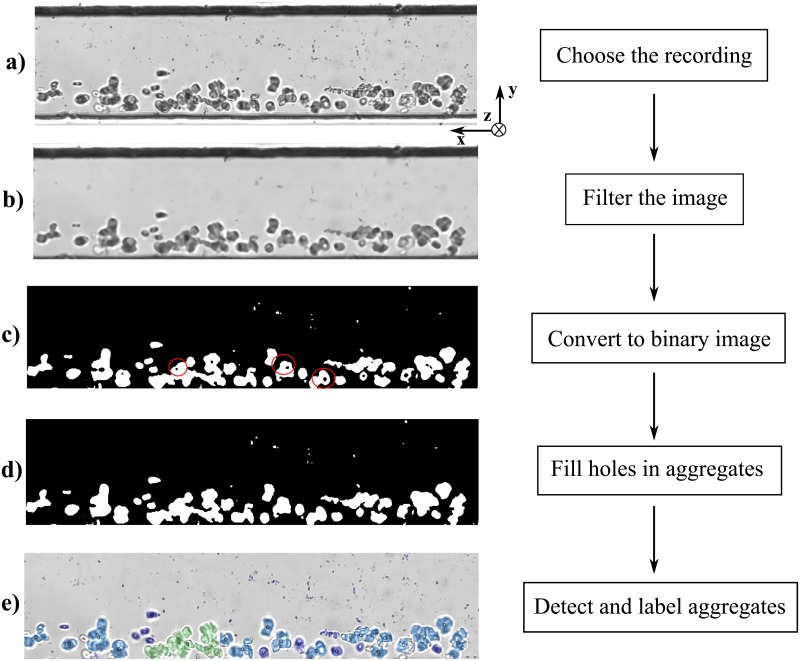
Flowchart of the MATLAB code for image processing of RBC aggregates flowing in microchannels. The red circles in the image show examples of the incomplete aggregate detection due to the non-uniform light distribution in the image.

For each test, the RBC aggregates flowing in the channel were recorded for 7 seconds, capturing 1200 frames. The field of view was 487 μm long, allowing for visualization of multiple aggregates at a time. The images recorded for each test were processed using a MATLAB program based on image pixel intensity. A flowchart illustrating the processing steps is shown in Fig 4, and full details of the image processing method and its validation are provided in Mehri et al. [42]. Each image in Fig 4 represents 1286 *×* 300 pixels^2^. The two-dimensional areas of the aggregates were measured for each frame to obtain an average aggregate size per frame; these, in turn, were averaged for the entire recording to obtain a global average aggregate size per recording. The areas detected in pixels were then converted to μm^2^ based on the conversion factor for the specific lens used. From these images, the distributions of RBCs within the aggregates were also analyzed. No distinction was made between the “rouleaux” and three dimensional cluster structures described in the introduction.

**Fig 5:**
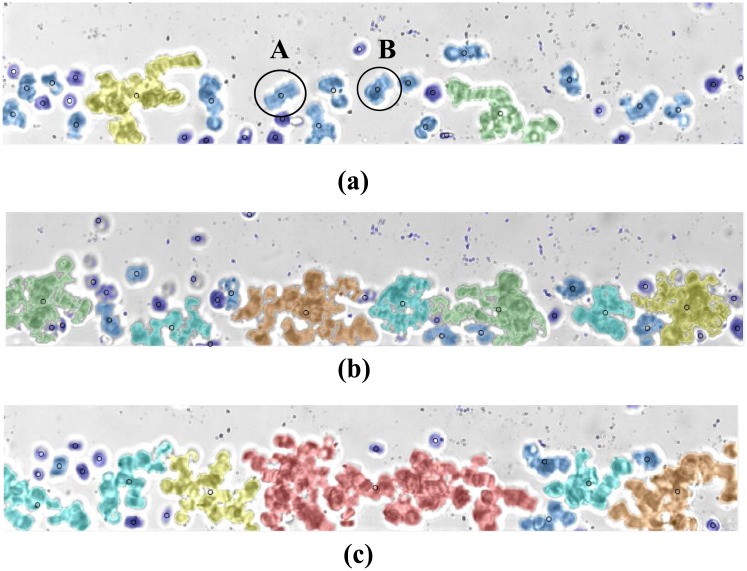
Processed images of human RBC aggregates flowing at 10 μL/hr suspended at (a) 5%, (b) 10% and (c) 15% H. The color coding is based on the sizes of the aggregates as shown in Fig 6A and 6B are two “rouleaux” present in the flow.

**Fig 6:**
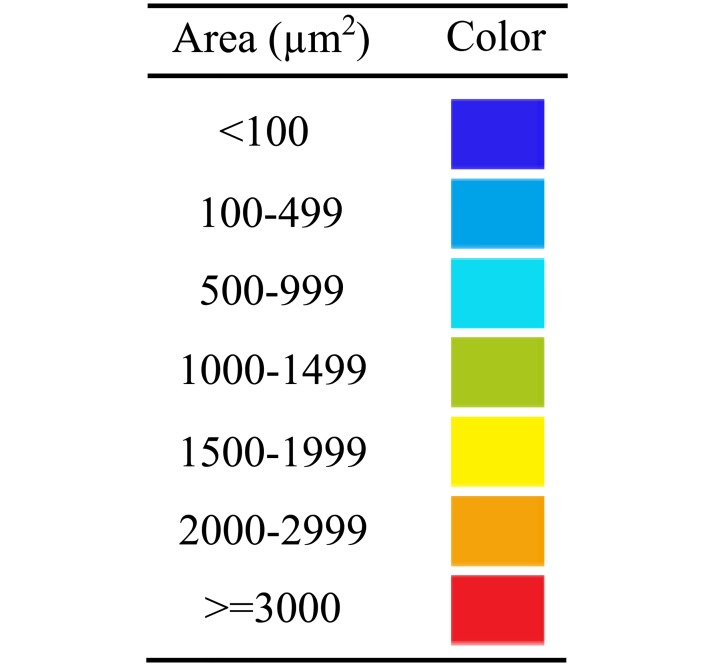
Color coding of the detected aggregates based on the aggregate sizes.

**Fig 7:**
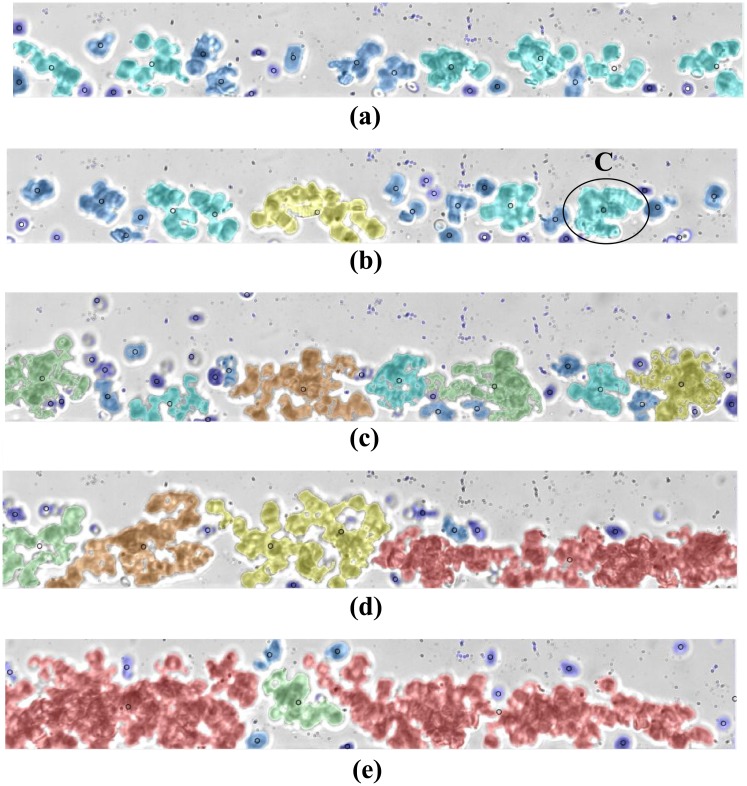
Processed images of human RBC aggregates suspended at 10% H, flowing at (a) 35 μL/hr, (b) 20 μL/hr, (c) 10 μL/hr, (d) 7.5 μL/hr and (e) 5 μL/hr. The color coding is based on the sizes of the aggregates as shown in Fig 6. C represents a three dimensional cell cluster present in the flow.

**Fig 8:**
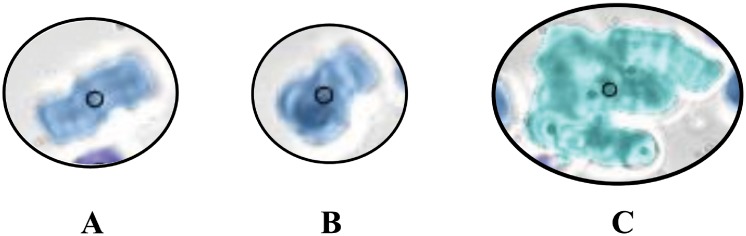
RBC aggregate shapes: “rouleaux” (A and B) as identified in Fig 5a at 5% H and three dimensional cell cluster (C) as identified in Fig 7b at 10%H.

RBC aggregates detected from sample #11 flowing at Q = 10 μL/hr are shown in Fig 5a, 5b and 5c for hematocrit of 5, 10 and 15% hematocrit respectively and labeled based on their respective sizes as shown in Fig 6. Fig 7a–7e show the RBC aggregates (sample #11) at 10% hematocrit flowing at 35, 20, 10, 7.5 and 5 μL/hr, respectively, labeled based on their respective sizes using image processing. Fig 8 shows an enlargement of the different aggregate shapes (“rouleaux” and three dimensional clusters) identified in the flow (sample #11).

### Viscosity measurements

The apparent viscosity of the blood suspension was measured in the microchannel using the concept of optical viscometers [24]. This concept is based on the analytical solution of two co-flowing fluids in a rectangular cross-section that can be obtained by a Fourier series expansion. For a rectangular channel of width *w* and depth *h* (Fig 2), the analytical solution of two co-flowing laminar streams of incompressible fluids in a rectangular channel can be used to obtain the flow rate ratio as a function of the viscosity ratio as follows [27]:

$$\frac{Q_{1}}{Q_{2}}=\frac{\mu_{1}}{\mu_{2}}\left(\frac{0.5+Y-\beta \sum_{n=1}^{\infty }\vartheta (\zeta -\kappa \xi \varphi )}{0.5-Y-\beta \sum_{n=1}^{\infty }\vartheta (\alpha -\xi \chi )}\right),$$ (5)

where:

$$\begin{array}{l} Y=\frac{1}{2}-\frac{w_{1}}{w},\\ \vartheta =48\frac{1-{\left(-1\right)}^{n}}{\pi^{5}n^{5}},\\ \zeta =\frac{\text{sinh}(2\pi nY/2\beta )}{\text{cosh}(n\pi /2\beta )}+\text{tanh}(n\pi /2\beta ),\\ \alpha =\frac{\text{sinh}(2\pi nY/2\beta )}{\text{cosh}(n\pi /2\beta )}-\text{tanh}(n\pi /2\beta ),\\ \kappa =\frac{\text{tanh}(2\pi nY/2\beta )\text{tanh}(n\pi /2\beta )-1}{\text{tanh}(2\pi nY/2\beta )\text{tanh}(n\pi /2\beta )+1},\\ \varphi =\text{sinh}(2\pi nY/2\beta )\text{tanh}(n\pi /2\beta )+\text{cosh}(2\pi nY/2\beta )-\frac{1}{\text{cosh}(n\pi /2\beta )},\\ \chi =\text{sinh}(2\pi nY/2\beta )\text{tanh}(n\pi /2\beta )+\text{cosh}(2\pi nY/2\beta )+\frac{1}{\text{cosh}(n\pi /2\beta )},\\ \xi =\frac{\left(\frac{\mu_{2}}{\mu_{1}}-1)(\frac{1}{\text{cosh}(2\pi nY/2\beta )}-\frac{1}{\text{cosh}(\pi n/2\beta )}\right)}{\left(\frac{\mu_{2}}{\mu_{1}}+\kappa )\text{tanh}(2\pi nY/2\beta )+(\kappa -\frac{\mu_{2}}{\mu_{1}})\text{tanh}(n\pi /2\beta \right)}. \end{array}$$

Here, *μ*_1_ is the viscosity of the reference fluid (PBS), *μ*_2_ is the viscosity of the test fluid (blood; in this case *μ*_2_ is an apparent viscosity), *Q*_1_ is the flow rate of the reference fluid, *Q*_2_ is the flow rate of the test fluid, *w*_1_ is the width of the reference fluid in the channel, *w*_2_ is the width of the test fluid in the channel (*w* = *w*_1_ + *w*_2_), *Y* is the dimensionless location of the interface and *β* is the channel aspect ratio denoted as *β = h/w*. For the known flow rates *Q*_1_ and *Q*_2_ in our double-Y microchannel device, we determined the apparent viscosity of the blood from Eq 5. The diffusion layer between the blood and the phosphate buffered saline was examined experimentally using fluoroscopy and shown to be minimal due to the small Reynolds number (Re = 0.03).

Plasma viscosity has been shown to be indicative of the molecular weight and molecular shape of the protein [43]. Therefore, plasma viscosity can be used as a controlling factor to compare the aggregation size and viscosity between samples. The plasma viscosity was measured using the m-VROC^*TM*^ viscometer (Rheosence Inc., USA) with temperature control. In order to obtain more control over the viscosity data, the apparent viscosity is divided by the donor-specific plasma viscosity to obtain the relative viscosity.

The experimental apparent viscosities measured using optical viscometry were fitted using a linear least squares method to a power law model, wherein the fluid consistency index *K* and the non-Newtonian behaviour index *n* were identified as the intercept and the slope of the regression, respectively. The experimental data were also fitted using a nonlinear least-squares solver using the Levenberg-Marquart method [44] to a Carreau model. The parameters, namely the infinite shear viscosity, the zero shear viscosity, the relaxation time, and the power law index *n* were extracted from the fitted curves. The coefficient of determination, R^2^, was calculated as a measure of the “goodness of fit”. It is important to note that the coefficient of determination is usually calculated for linear least squares fits and was shown to be less relevant for non-linear least squares models, despite its extensive use in biochemical research [45]. For this purpose, the root mean square error (RMSE) is additionally presented in Tables 1–6, and is calculated as follows:

$$RMSE=\sqrt{\frac{\sum_{j=1}^{m}{\left(\mu_{\exp,j}-\mu_{fit,j}\right)}^{2}}{m-p}},$$ (6)

where *μ*_*exp*,j_ represents the experimental data point *j* of viscosity, *μ*_*fit*,j_ the fitted data point *j* of viscosity, *m* the number of data points and *p* the number of parameters to be determined from the fitted curve (2 for the power law model and 4 for the Carreau model). The RMSE estimates the standard deviation of the random component of the data. This measure is intuitive, as it has the same units as the fitted variable, however, because it is relative to the absolute viscosity measurement, it is not always appropriate to compare fits for data of different conditions, having different absolute viscosity values. For this reason, and because of its widespread familiarity, the coefficients of determination for the different hematocrits are still provided in this study for comparison purposes.

**Table 1:** Summary of the non-Newtonian parameters for the power law model for human blood suspended at 5%, 10% and 15% H at 23°*C* with the corresponding R^2^ and RMSE.

Hematocrit (%)	*K* (Pa.s^n^)	*n*	R^2^	RMSE (cP)
5	9.9	0.603	0.33	1.81
10	50.9	0.156	0.80	4.93
15	62.2	0.197	0.78	3.62

**Table 2:** Summary of the non-Newtonian parameters for the Carreau model for human blood suspended at 5%, 10% and 15% H at 23°C with the corresponding R^2^ and RMSE.

Hematocrit (%)	*μ*_*0*_ (cP)	*μ*_*∞*_ (cP)	*λ* (s)	*N*	R^2^	RMSE (cP)
5	26.9	1.6	3.313	0.353	0.33	1.85
10	89.9	1.6	3.312	0.369	0.62	4.98
15	118.6	2.3	3.312	0.362	0.80	3.85

**Table 3:** Summary of the non-Newtonian parameters for the power law model for human blood suspended at 5%, 10% and 15% H fit to the apparent viscosity data, with the corresponding R^2^ and RMSE at 37°C. The parameters currently proposed in the literature for blood at 45% H tested at 37°C are provided for reference.

Hematocrit (%)	*K* (Pa.s^n^)	*n*	R^2^	RMSE (cP)
5	9.3	0.660	0.58	1.10
10	10.8	0.680	0.64	1.40
15	17.1	0.568	0.22	4.31
45	35.0	0.600	Cho and Kensey [32]	
45	17.0	0.708	Shibeshi and Collins [33]	

**Table 4:** Summary of the non-Newtonian parameters for the Carreau model for human blood suspended at 5%, 10% and 15% H fit to the apparent viscosity data, with the corresponding R^2^ and RMSE at 37°C. The parameters currently proposed in the literature for blood at 45% H tested at 37°C are provided for reference.

Hematocrit (%)	*μ*_*0*_ (cP)	*μ*_*∞*_ (cP)	*λ* (s)	*N*	R^2^	RMSE (cP)
5	23.3	2.0	3.313	0.352	0.52	1.06
10	26.4	2.6	3.312	0.353	0.52	1.47
15	139.5	4.2	3.314	-0.152	0.64	3.49
45	56.0	3.45	3.313	0.356	Cho and Kensey [32]	

**Table 5:** Summary of the non-Newtonian parameters for the power law model for human blood suspended at 5%, 10% and 15% H fit to the relative viscosity data, with the corresponding R^2^ and RMSE at 37°C. The values for *K* are given as relative values to the plasma viscosity *μ*_*p*_.

Hematocrit (%)	*K* / *μ*_*p*_ (s^n-1^)	*n*	R^2^	RMSE / *μ*_*p*_
5	5.6	0.611	0.62	0.50
10	6.2	0.646	0.75	0.59
15	8.7	0.586	0.22	2.47

**Table 6:** Summary of the non-Newtonian parameters for the Carreau model for human blood suspended at 5%, 10% and 15% H, fit to the relative viscosity data, with the corresponding R^2^ and RMSE at 37°C. The values of *μ*_*0*_, *μ*_*∞*_ and RMSE are given as relative values to the plasma viscosity *μ*_*p*_.

Hematocrit (%)	*μ*_*0*_ / *μ*_*p*_	*μ*_*∞*_ / *μ*_*p*_	*λ* (s)	*n*	R^2^	RMSE / *μ*_*p*_
5	14.1	0.9	3.313	0.352	0.64	0.51
10	15.3	1.2	3.313	0.353	0.68	0.62
15	27.3	1.0	3.314	0.354	0.42	2.35

## Results

### Room temperature results

Healthy and fresh human blood samples obtained from six volunteers (*n*_*samples*_ = 6) were used for the experiments conducted at 23°C. It is important to note that, for the different hematocrits, RBCs were suspended in their native plasma where protein concentration varied from one sample to another, hence causing some natural variation in aggregation. All the RBC suspensions were tested following the procedure outlined in the experimental procedure section.

**Fig 9:**
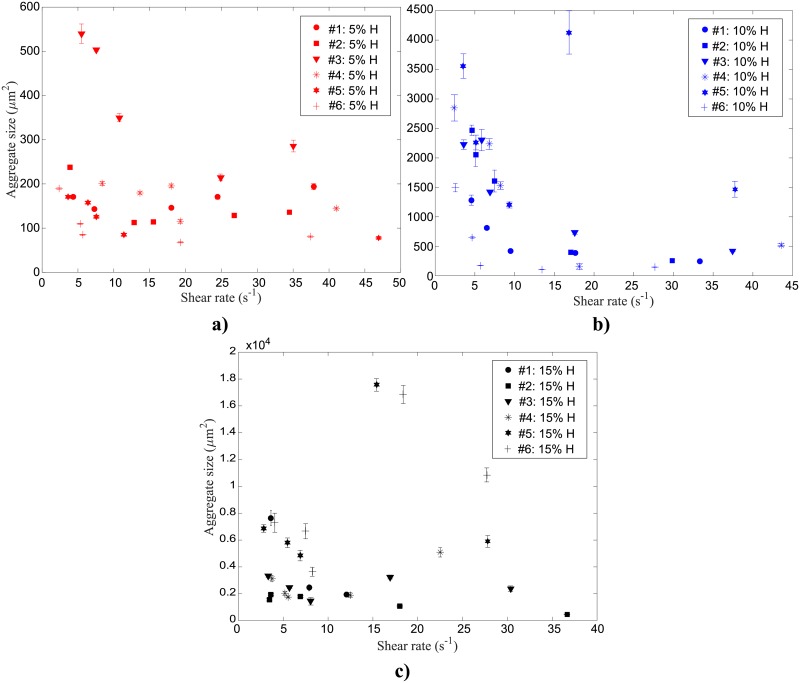
Average human RBC aggregate sizes for six different blood samples suspended at (a) 5% (b) 10% and (c) 15% H as a function of shear rate at 23°*C*. Error bars for each sample are provided for the standard error of several (2–5) tests performed consecutively.

In Fig 9a, 9b and 9c the average aggregate sizes are plotted as a function of shear rate for each sample 5%, 10%, and 15%H, respectively. Error bars shown for each measurement depict the standard error obtained when averaging the results of several tests (2–5) performed consecutively under the same conditions.

The results show that for the 5% H suspensions (Fig 9a), changes in shear rates do not strongly influence the aggregate sizes detected. The aggregate sizes, for all the shear rate values, ranged from approximately 100 to 550 μm^2^. For the 10% H samples (Fig 9b), for larger shear rates (>10 s^-1^), aggregate sizes ranged from approximately 200 to 1500 μm^2^, whereas for shear rates less than 10 s^-1^, the aggregate sizes were as large as 3500 μm^2^. Increasing the hematocrit to 15% (Fig 9c), the aggregate sizes increased dramatically, typically ranging from 1500 to 8000 μm^2^ for the same shear rates. The trend for the 10% H case is also observed for 15% H, in which the average aggregate size increases for shear rates smaller than 10 s^-1^, although the trend is not as pronounced.

**Fig 10:**
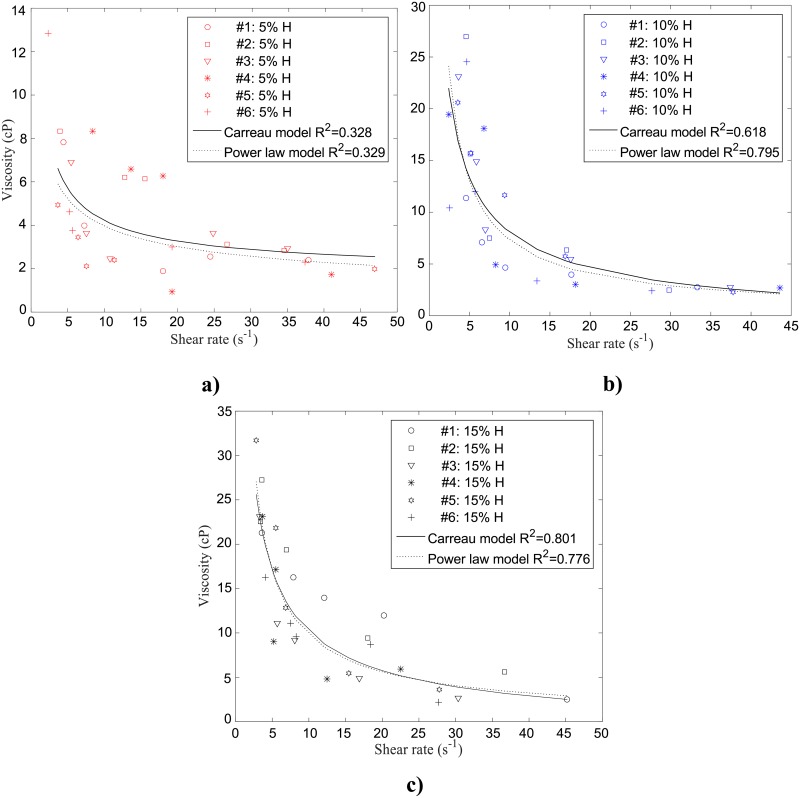
Viscosity measurement for six different human blood samples suspended at (a) 5% (b) 10% and (c) 15% H as a function of shear rate at 23°*C*. Curve fittings of power law (dotted black curve) and Carreau (solid black curve) models are also shown with the associated R^2^ value.

The measured viscosities are plotted in Fig 10a, 10b and 10c, as a function of shear rate for each of the three hematocrits (5%, 10% and 15% H, respectively). As expected, viscosity is quite large for low shear rates (< 10 s^-1^) tailing off to lower values at higher shear rates for all three hematocrits studied. The trend is more pronounced for the 10% and 15%H. Tables 1 and 2 summarize the parameters obtained for the power law and Carreau models, respectively, for the different hematocrits with the corresponding R^2^ and RMSE values. The viscosity data were better represented by the power law and Carreau models for the higher hematocrits (Fig 10b and 10c), as depicted by the coefficients of determination shown in Tables 1 and 2. For 5% H, the videos of the flow confirmed that the flow of aggregates was more variable than in the 10% H case, since there were so few and they sometimes would get stuck in the entrance or suddenly get released.

### Body temperature results

**Fig 11:**
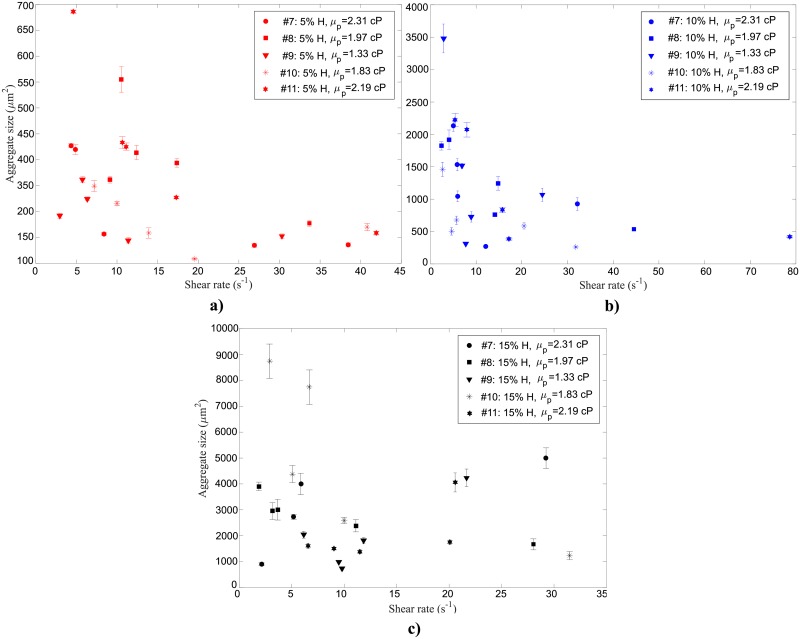
Average RBC aggregate sizes for five different human blood samples suspended at (a) 5% (b) 10% and (c) 15% H as a function of shear rate at 37°C. Error bars for each sample are provided for the standard error of several (2–5) tests performed consecutively. The plasma viscosity *μ*_*p*_ is provided for each sample.

**Fig 12:**
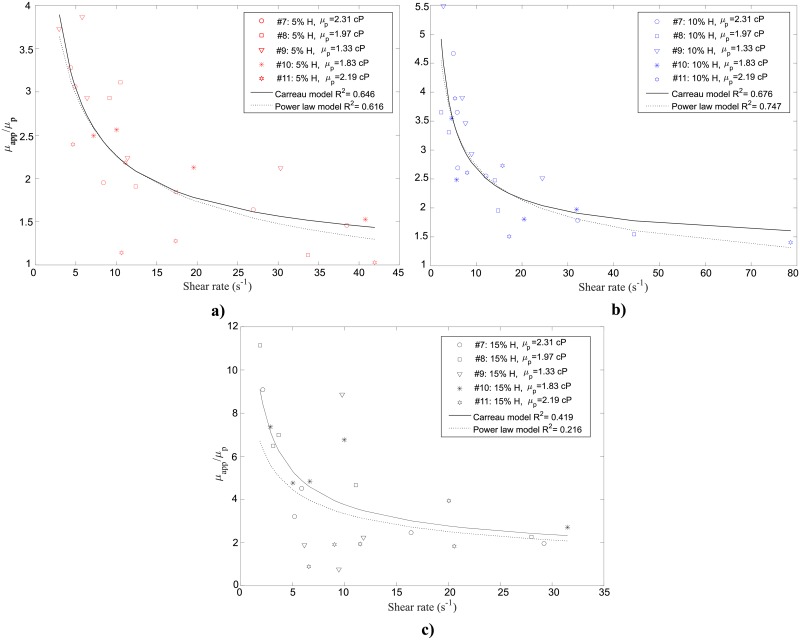
Relative viscosity measurement for five different human blood samples suspended at (a) 5% (b) 10% and (c) 15% H as a function of shear rate at 37°C. Curve fittings of power law (dotted black curve) and Carreau (solid black curve) models are also shown with the associated R^2^ value. The plasma viscosity *μ*_*p*_ is provided for each sample.

Fresh human blood samples, obtained from five healthy volunteers (*n*_*samples*_ = 5), were used for the experiments at 37°C. As was done for the tests performed at room temperature, all the RBC suspensions were tested following the procedure detailed in the experimental procedure section. Fig 11a, 11b and 11c present the average RBC aggregate sizes measured *vs*. shear rate for 5%, 10%, and 15% H, respectively at 37°C. Error bars shown for each measurement depict the standard error obtained when averaging the results of several tests performed consecutively under the same conditions. The plasma viscosity *μ*_*p*_ is provided in the Figs 11 and 12 for reference.

At 37°C, the average RBC aggregate sizes found for the 5% H blood samples in Fig 11a do not vary greatly with the shear rate, as was observed for the room temperature experiments. The samples that present higher average aggregate sizes (159–586 μm^2^) for different shear rates (4.6–41.9 s^-1^) have a relatively high plasma viscosity (e.g. *μ*_*p #11*_ = 2.19 ±0.13 cP). For the sample having the lowest plasma viscosity (*μ*_*p #9*_ = 1.33 ± 0.05 cP) lower average aggregate sizes (145–262 μm^2^) were found for the varying shear rates (2.9–30.3 s^-1^). These results suggest that higher plasma viscosities correlate with larger aggregate sizes, which may be explained by them having higher levels of proteins that promote aggregation. For the 10% H suspensions (Fig 11b), the average aggregate size does vary with the shear rate and increases especially at low shear rates (*<* 10 s^-1^). However, for intermediate shear rates (10–30 s^-1^), there was a relatively large spread in the measured aggregate sizes. As was found for the 5% H suspensions, the larger aggregation sizes corresponded to the samples with higher plasma viscosities (*μ*_*p #7*_ = 2.31± 0.08 cP and *μ*_*p #11*_ = 2.19 ± 0.13 cP). No clear trend could be detected for the 15% H suspensions due to the difficulty in distinguishing separate aggregates among the compact RBCs.

The measured apparent viscosity of the suspensions is normalized by the donor-specific plasma viscosity to obtain the relative viscosity *μ*_*relative*_, shown as a function of shear rate in Fig 12a, 12b and 12c for 5%, 10% and 15% H, respectively. As for the room temperature results of apparent viscosity, high relative viscosity values were obtained at lower shear rates (< 10 s^-1^). The viscosity was found to decrease for larger shear rates, depicting the shear-thinning blood behaviour.

Tables 3 and 4 summarize the non-Newtonian parameters obtained for the power law and Carreau models, respectively, fit to the apparent viscosity data, along with the corresponding R^2^ and RMSE values. Both fits for the suspensions at 5% H were found to be moderate (R^2^ = 0.58 and R^2^ = 0.52 for power law and Carreau models respectively). Increasing the hematocrit to 10% and 15% H slightly improves the corresponding coefficients of determination for the power law fit of the 10%H and the Carreau fit of the 15%H. The highest R^2^ (0.64) were found to correspond to the fitted curve of the power law model at 10% H and the Carreau model for the 15% H suspensions. However, the viscosity data at 15% H were not well represented by the power law model.

These results differ from the results obtained at room temperature, where better fits of the Carreau and power law model were found at 10% and 15% H: i.e. a larger spread of data between the samples was obtained at body temperature.

Tables 5 and 6 summarize the normalized non-Newtonian parameters obtained for the power law and Carreau models, respectively, fit to the relative viscosity data, along with the corresponding R^2^ and normalized RMSE. For the 5% H and 10% H suspensions, good representation by both models was obtained for the relative viscosity. For the 15% H suspensions, the relative viscosity variation is not well represented by either the power law or Carreau models (Tables 5 and 6).

**Fig 13:**
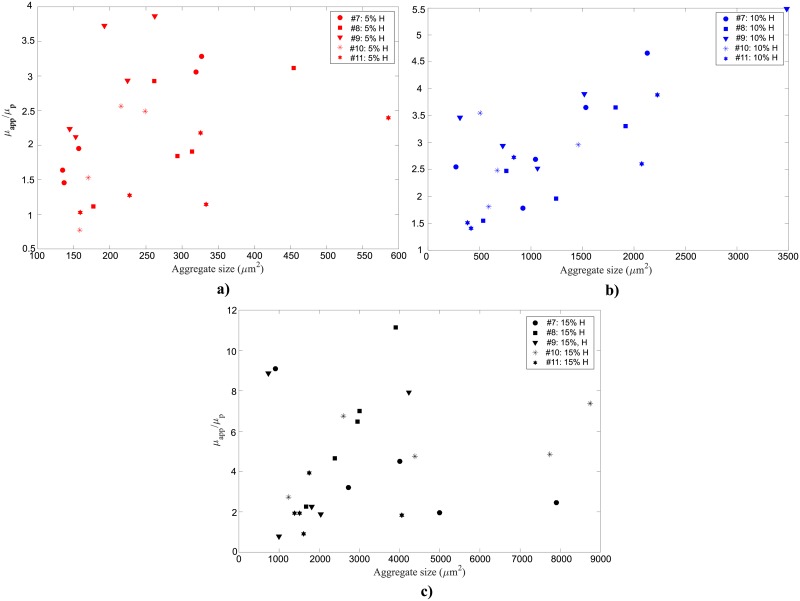
Relative viscosity measurement for five different human blood samples suspended at (a) 5% (b) 10% and (c) 15% H as a function of average aggregate size at 37°C.

Fig 13a, 13b and 13c present the relative viscosity as a function of average aggregate size for 5%, 10% and 15% H, respectively at 37°C. The trend for the combined samples shows that an increase in the aggregate sizes results in an increase in viscosity. Increase in viscosity per unit increase in aggregate size (i.e. the slope of the fitted data) varies not only from sample to sample but also with hematocrit. In fact, an increase in blood hematocrit results in a smaller degree of viscosity increase per unit change of aggregate size.

### Temperature effect

**Fig 14:**
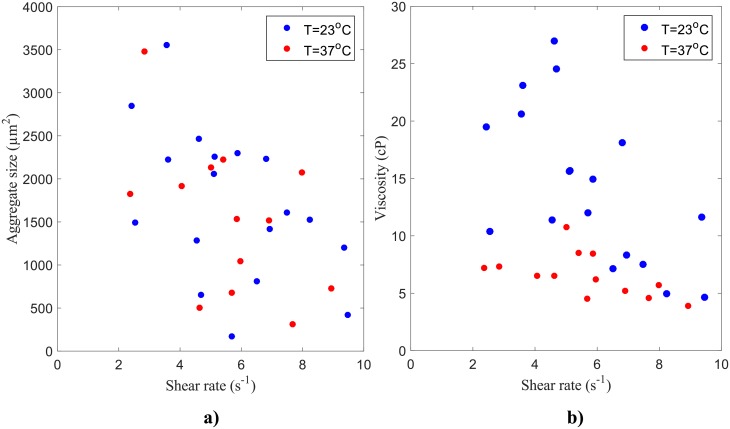
Variation of (a) RBC aggregate size and (b) apparent viscosity with shear rate for all 10% H tests by temperature: 23°C (blue) and 37°C (red).

In order to assess the effect of the temperature on the blood samples, the average aggregate sizes (Fig 13a) and the viscosity of the blood (Fig 14b) at 10% H were plotted as a function of the shear rate for both temperatures 23°C and 37°C. An increase in viscosity is noted for the lower temperature. These findings agree with previous studies [46, 47]. Fig 14a shows slightly smaller aggregate sizes at higher temperatures, however, no clear correlation was observed.

## Discussion

### Effect of shear rate and hematocrit on RBC aggregation in microcirculation

The average RBC aggregates, measured in the two fluid-flow shear flow microchannel system, were quantified for different shear rates and for three low hematocrits at different temperatures. It was found that the shear rate did not strongly influence the aggregate sizes at 5% H at both temperatures. At 10% H, an increase of the aggregate sizes is observed for shear rates below 10 s^-1^. Higher sample plasma viscosity also correlated with higher aggregate sizes. At 15% H, the aggregates were shown to increase at lower shear rates. However, no clear trend could be detected for the 15% H suspensions due to the large number of cells in the field of view.

The sample hematocrit was also shown to influence the average aggregate sizes where an increase in hematocrit resulted in an increase in aggregate size. The results obtained agree with the conclusion of Kim et al. [48, 49], who investigated the link between the collision rate of erythrocytes and the degree of aggregation *in vivo* noting a hematocrit of 9%. The number of RBCs present within the medium greatly affects the collision rate since the fewer the number of cells, the lower the probability of the RBCs to encounter, collide and initiate rouleaux formation, thereby decreasing aggregation [20]. The findings of this study provide quantitative data on the average aggregate sizes in microcirculation under different conditions.

Although a trend can be seen when considering all the samples in Figs 9 and 10, discrepancies between the different blood samples are clearly present. This is mainly due to the physiological nature of blood varying from one individual to another. A predominant source of spread in the results is likely the variation in fibrinogen concentration in the plasma. Fibrinogen is also known to affect the aggregation mechanism and varies from person to person. Plasma viscosity has been shown to be indicative of the molecular weight and molecular shape of the protein [43], and can therefore be used as another influencing factor to compare the aggregation size and viscosity between samples. Plasma viscosity, however, is not directly linked to fibrinogen concentration and therefore cannot fully explain the differences between the samples [43].

### Effect of shear rate and hematocrit on blood viscosity in microcirculation

Viscosity measurements, performed using the optical viscometer technique, were shown to be shear rate dependent with a shear-thinning behaviour. The viscosity of blood was found to increase with decreasing shear rate. The viscosity was also found to increase with increasing the hematocrit, especially at low shear rates. This shear-thinning behaviour was expected and has been extensively reported [15, 20–22]. The non-Newtonian parameters obtained by fitting the viscosity data to the power law and Carreau models showed different behaviours at different hematocrits and temperatures. For the power law model, the consistency index (*K*) was found to increase with increasing the hematocrit at both temperatures. The power law index (*n*) values indicate a more prominent shear-thinning behaviour at higher hematocrits at room temperature. At body temperature, the power law index remains nearly constant showing similar shear-thinning behaviour between hematocrits. The power law parameters obtained at low hematocrits in microcirculation were compared to the parameters currently used in the literature [32, 33] at 45% H (Table 3). As observed in Table 3, the power law parameters available in the literature do vary for similar hematocrits. The results obtained at 15% H approach the parameters reported by Shibeshi and Collins [33] at 45% H while differing from the parameters used in the study of Cho and Kensey [32]. However, a low coefficient of determination was reported for the power law fit at 15% H.

The non-Newtonian parameters obtained by fitting the viscosity data to the Carreau model, were found to better represent the data. For the temperatures tested, the viscosity at zero shear (*μ*_*0*_) and the viscosity at infinite shear (*μ*_*∞*_) increase with increasing hematocrit. The parameters obtained at body temperature were also compared to the parameters reported in the study of Cho and Kensey [32]. At 15%H, the larger spread of data between the samples at body temperature made the results inconclusive. Different samples used exhibited differences in their specific non-Newtonian behaviours, producing significant variations in viscosity from one sample to another. These non-Newtonian models better represent the viscous behaviour of blood when fitted for each individual subject, instead of fitted to the ensemble data. For example, there was significant variation in the plasma viscosities measured for the different subjects (between 1.33 and 2.31 cP). Measurements reported by Mehri [50] revealed that the plasma viscosity had a strong influence on the apparent viscosity of RBC suspensions at low shear rates (<10 s^-1^), with a diminishing influence at higher shear rates. While we recognize that individual blood samples vary in their exact viscous behaviour, we report here the model parameters for the data fitted as an ensemble, in order to provide parameters representative of average blood for future use. The parameters obtained from the fitted curves can be used for simple modeling of the non-Newtonian behaviour of blood in microcirculation.

### Effect of RBC aggregation on blood viscosity

It is clear from our data that viscosity increases with RBC aggregate size. Our study of three hematocrits shows that as hematocrit increases, the rate of increase of viscosity per unit increase of aggregate size does not necessarily follow a linear trend, as previously reported by Kaliviotis and Yianneskis [34]. Nor would we expect it to due to the complex interaction of irregularly shaped aggregates. This relationship could be due to differences in the structural integrity of the RBC network: Aggregates were observed to form and break apart under shear which would affect the apparent viscosity measured. However, a systematic study was not performed. A better understanding of the molecular interaction of the aggregates is required in order to interpret the results with certitude.

### Temperature effects on RBC aggregate size and blood viscosity

The same trends were found for both the results obtained at room temperature and body temperature, with lower viscosities measured at body temperature as shown in Fig 14a and 14b. It is well known that temperature greatly affects blood behaviour [20]. The temperature effect is commonly reported in viscosity measurements, however no quantification of the temperature effect has been provided in terms of aggregate size. Neumann et al. [46] did show the temperature effects on RBC aggregation and stated that a decrease in temperature engenders a higher resistance of the aggregates to hydrodynamic dispersion and hence would increase the absorption energy of the aggregates due to an increase in molecular adsorption stress. However, this temperature dependence was not directly related to RBC aggregate size. The results of the present investigation confirm the conclusions of Neumann et al. [46]: significantly higher viscosities were measured at low shear rates for the room temperature results than for the body temperature results, as shown in Fig 14b, however no correlation for aggregate size with temperature was observed (Fig 14a).

### Limitations

For this study, the control of several parameters was essential in order to assess the sizes of the RBC aggregates in a controlled microfluidic environment. However, parameter variation was inevitable and is discussed in this section.

The reported hematocrit in this study represents the sample hematocrit at the entrance of the feeding tube. Although the hematocrit at the entrance of the microchannel is controlled, variations in local hematocrit were observed. These variations were expected as observed in *in vivo* studies [51]. In order to account for the hematocrit variation within the microchannel, the aggregate sizes are reported as an average of multiple consecutives frames.

Different hematocrits, up to 15%, were analyzed in this study. Higher hematocrits were not further analyzed due to the optical system limitations. As previously noted, difficulties were encountered in the visualization of the aggregate at 15% at lower shear rates. Therefore, assessing the sizes of the aggregates at higher hematocrits (up to 20–25% in the microcirculation) would require further investigation. It is hypothesized that combining the methodology used in this study with a confocal microscope could provide further understanding of aggregate formation in three dimensions. However, this would require analyzing the aggregate in a “freeze-frame” manner in order to reconstruct the aggregates in three dimensions which is extremely challenging with a dynamic flow.

Due to the depth of the microchannel (allowing the formation of aggregates with no constraint), overlapping of aggregates flowing in different planes in the microchannel was visualized which could affect the sizes of the aggregates detected. In order to remedy the problem, the aggregates were detected on multiple consecutive frames where the aggregate sizes are averaged in time and space hence reducing the effect of aggregate overlap.

In this study, we considered only steady flows. However, *in vivo*, blood flow is pulsatile. Intuitively, due to the unsteady nature of the flow, it is believed that the pulsatile flow could affect the blood viscosity and aggregation, however this was not investigated in this study. The purpose of the study was to investigate blood behaviour and red blood cells aggregation in microcirculation, under controlled flow conditions. In order to provide controlled conditions for shear rate and aggregate size measurements, a steady flow was required. In microcirculation, the effects of pulsatile flow are minor [52] and the microcirculatory network represents a relatively compliant system [53].

In this study, two of the more common models used to model blood flows in complex geometries were used to fit the apparent viscosity data. However, these models do not consider hematocrit or RBC aggregation. Several other models have attempted to mimic hematocrit dependence and yield stress behaviour at the macroscale [14–16]. In order to obtain an accurate model mimicking the microrheological behaviour of blood at low hematocrits further investigation is required. We provided non-Newtonian parameters at low hematocrits for the common models for future use of simple models and to validate our results to some degree since there is little data available to compare.

## Conclusion

This research investigated blood behaviour in a controlled microfluidic system for varying shear rate, three different hematocrits, at both room and body temperatures. Human RBC aggregate sizes were quantified and plotted as a function of corresponding shear rates at different hematocrits. The results give quantitative effects of temperature, hematocrit, shear rate and viscosity on RBC aggregate sizes. The viscosity data were fitted to two simple empirical models that were previously developed to analyze the non-Newtonian behaviour of polymers: the power law model and the Carreau model. The non-Newtonian parameters associated with these models were determined by fitting the experimental data and can be used towards the simple modeling of blood’s non-Newtonian behaviour in microcirculation.

This work provides important contributions to the understanding of blood behaviour in microcirculation and the hemorheology field. The results obtained at room temperature constitute a framework for the design of lab-on-chip devices and artificial organs on chips. Overall, the results obtained provide a deeper understanding of human RBC aggregates in microcirculation by quantifying aggregate sizes and associated viscosity variation at low shear rates and could help to determine aggregate behaviour in clinical settings.

## References

[1] Fåhraeus R, Lindqvist T. The viscosity of the blood in narrow capillary tubes. Am J Physiol—Legacy Content. 1931;96: 562–568.

[2] Pries AR, Neuhaus D, Gaehtgens P. Blood viscosity in tube flow: dependence on diameter and hematocrit. Am J Physiol Heart Circ Physiol. 1992;263: H1770–H1778. 10.1152/ajpheart.1992.263.6.H1770 1481902

[3] Cokelet GR, Goldsmith HL. Decreased hydrodynamic resistance in the two-phase flow of blood through small vertical tubes at low flow rates. Circ Res. 1991;68: 1–17. 10.1161/01.RES.68.1.1 1984854

[4] Perkkio J, Keskinen R. Hematocrit reduction in bifurcations due to plasma skimming. Bull Math Biol. 1983;45: 41–50. 10.1016/S0092-8240(83)80040-8 6850160

[5] Merrill EW, Gilliland ER, Lee TS, Salzman EW. Blood Rheology: Effect of Fibrinogen Deduced by Addition. Circ Res. 1966;18: 437–446. 4952703 10.1161/01.res.18.4.437

[6] Chien S, Usami S, Taylor HM, Lundberg JL, Gregersen MI. Effects of hematocrit and plasma proteins on human blood rheology at low shear rates. J Appl Physiol. 1966;21: 81–87. 10.1152/jappl.1966.21.1.81 5903948

[7] Skalak R, Keller SR, Secomb TW. Mechanics of blood flow. J Biomech Eng. 1981;103: 102–115. 10.1115/1.3138253 7024641

[8] Cokelet GR, Merrill EW, Gilliland ER, Shin H. The rheology of human blood—Measurement near and at zero shear rate. J Rheol. 1963;7: 303–317. 10.1122/1.548959

[9] Reiner M. Rhe´ologie The´orique. Paris: Dunod; 1955.

[10] Pries AR, Secomb TW. Rheology of the microcirculation. Clin Hemorheol Microcirc. 2003;29: 143–148. 14724335

[11] Casson N. A flow equation for pigment-oil suspensions of the printing ink type Rheology of Disperse Systems 1959.

[12] Carreau PJ. Rheological equations from molecular network theories. Trans Soc of Rheol. 1972;16: 99–127. 10.1122/1.549276

[13] Yasuda K. Investigation of the analogies between viscometric and linear viscoelastic properties of polystyrene fluids. Ph.D. Thesis, Massachusetts Institute of Technology. 1979.

[14] Caton F. The sol-gel transition of blood. Presented at the Workshop Agregation érythrocytaire & ecoulement sanguine, Marseille, France. 2015.

[15] Walburn FJ, Schneck DJ. A constitutive equation for whole human blood. ASME Bioengineering Division. Winter Annual Meeting; 1975.

[16] Herschel W, Bulkley R. Konsistenzmessungen von gummi-benzollösungen. Colloid Polym Sci. 1926;39: 291–300.

[17] Zhang JB, Kuang ZB. Study on blood constitutive parameters in different blood constitutive equations. J Biomech. 2000;33: 355–360. 10673119 10.1016/s0021-9290(99)00101-3

[18] Cabel M, Meiselman HJ, Popel AS, Johnson PC. Contribution of red blood cell aggregation to venous vascular resistance in skeletal muscle. Am J Physiol. 1997;272: H1020–32. 10.1152/ajpheart.1997.272.2.H1020 9124410

[19] Bishop JJ, Nance PR, Popel AS, Intaglietta M, Johnson PC. Effect of erythrocyte aggregation on velocity profiles in venules. Am J Physiol Heart Circ Physiol. 2001;280: H222–H236. 10.1152/ajpheart.2001.280.1.H222 11123237

[20] Baskurt OK, Neu B, Meiselman HJ. Red Blood Cell Aggregation. Boca Raton: CRC Press; 2011.

[21] Bureau M, Healy JC, Bourgoin D, Joly M. Rheological hysteresis of blood at low shear rate. Biorheology 1980;17: 191–203. 6157430 10.3233/bir-1980-171-221

[22] Owens RG. A new microstructure-based constitutive model for human blood. J Non-Newtonian Fluid Mech. 2006;140: 57–70. 10.1016/j.jnnfm.2006.01.015

[23] Fedosov DA, Wenxiao P, Caswell B, Gompper G, Karniadakis GE. Predicting human blood viscosity in silico. Proc Natl Acad Sci USA. 2011;108: 11772–11777. 10.1073/pnas.1101210108 21730178 PMC3141939

[24] Chevalier J, Ayela F. Microfluidic on chip viscometers. Rev Sci Instrum. 2008;79: 076102-1-076102-3. 10.1063/1.2940219 18681739

[25] Galambos P, Forster F. An optical micro-fluidic viscometer. Int Mech Eng Cong Exp (Anaheim, CA: Dynamic Systems and Control Division, ASME). 1998;66: 187–191.

[26] Guillot P, Panizza P, Salmon JB, Joanicot M, Colin A, Bruneau CH et al Viscosimeter on a microfluidic chip. Langmuir. 2006;22: 6438–6445. 10.1021/la060131z 16800711

[27] Solomon DE, Vanapalli SA. Multiplexed microfluidic viscometer for high-throughput complex fluid rheology. Microfluid Nanofluidics. 2014;16: 677–690. 10.1007/s10404-013-1261-2

[28] Kaliviotis E, Dusting J, and Balabani S. Spatial variation of blood viscosity: Modelling using shear fields measured by a μPIV based technique. Med Eng Phys. 2011;33: 824–831. 10.1016/j.medengphy.2010.09.004 20943426

[29] Dusting J, Kaliviotis E, Balabani S, and Yianneskis M. Coupled human erythrocyte velocity field and aggregation measurements at physiological haematocrit levels. J Biomech. 2009;42: 1438–1443. 10.1016/j.jbiomech.2009.04.004 19428015

[30] Sherwood JM, Kaliviotis E, Dusting J, and Balabani S. Hematocrit, viscosity and velocity distributions of aggregating and non-aggregating blood in a bifurcating microchannel. Biomech Model Mechanobiol. 2014;13: 259–273. 10.1007/s10237-012-0449-9 23114881

[31] Chen S, Barshtein G, Gavish B, Mahler Y, and Yedgar S. Monitoring of red blood cell aggregability in a flow-chamber by computerized image analysis. Clin Hemorheol Microcirc. 1994;14: 497–508. 10.3233/CH-1994-14405

[32] Cho YI, Kensey KR. Effects of the non-Newtonian viscosity of blood on flows in a diseased arterial vessel. Part 1: Steady flows. Biorheology. 1991;28: 241–262. 1932716 10.3233/bir-1991-283-415

[33] Shibeshi SS, Collins WE. The rheology of blood flow in a branched arterial system. Appl Rheol. 2005;15: 398–405. 16932804 10.1901/jaba.2005.15-398 PMC1552100

[34] Kaliviotis E, Yianneskis M. On the effect of microstructural changes of blood on energy dissipation in Couette flow. Clin Hemorheol Microcirc. 2008;39: 235–242. 10.3233/CH-2008-1087 18503131

[35] Cheung YK, Shiovitz D, Sia SK. Microfluidic-based lithography for fabrication of multicomponent biocompatible microstructures In: Herold KE, Rasooly A, editors. Lab on a Chip Technology: Fabrication and Microfluidics. Norfolk: Caister Academic Press; 2009 pp 115–124.

[36] Mehri R, Laplante J, Mavriplis C, Fenech M. Investigation of blood flow analysis and red blood cell aggregation. J Med Biol Eng. 2014;34: 469–474. 10.5405/jmbe.1695

[37] Mehri R, Mavriplis C, Fenech. Design of a microfluidic system for red blood cell aggregation investigation. J Biomech Eng, M. 2014;136: 064501 10.1115/1.4027351 24700377

[38] Abdollahzadeh Jamalabadi MY, Daqiqshirazi M, Nasiri H, Safaei MR, Nguyen TK (2018) Modeling and analysis of biomagnetic blood Carreau fluid flow through a stenosis artery with magnetic heat transfer: A transient study. PLoS One. 2018;13: e0192138 10.1371/journal.pone.0192138 29489852 PMC5830309

[39] Vijayaratnam PRS, O’Brien CC, Reizes JA, Barber TJ, Edelman ER (2015) The impact of blood rheology on drug transport in stented arteries: steady simulations. PLoS One. 2015;10(6): e0128178 10.1371/journal.pone.0128178 26066041 PMC4466567

[40] Mehri R, Mavriplis C, Fenech M. Controlled microfluidic environment for dynamic investigation of red blood cell aggregation. J Vis Exp. 2015;100: e52719 10.3791/52719 26065667 PMC4545193

[41] Pitts K, Mehri R, Fenech M, Mavriplis C. Micro-particle image velocimetry measurement of blood flow: Validation and analysis of data pre-processing and processing methods. Meas Sci Technol. 2012;23: 105302 10.1088/0957-0233/23/10/105302

[42] Mehri R, Niazi E, Mavriplis C, Fenech M. An automatic method for dynamic Red Blood Cell aggregate detection in microfluidic flow. Physiol Meas. 2018;39: 01NT02 10.1088/1361-6579/aaa0ad 29227278

[43] Ke´sma´rky G, Kenyeres P, Ra´bai M, To´th K. Plasma viscosity: A forgotten variable. Clin Hemorheol Microcirc. 2009;41: 243–246. 10.3233/CH-2008-1088 18503132

[44] More´ JJ. The Levenberg-Marquardt algorithm: Implementation and theory In: Watson GA, editors. Numerical Analysis: Lecture notes in Mathematics vol 630 Berlin, Heidelberg: Springer; 1977 pp 105–116.

[45] Spiess A, Neumeyer N. An evaluation of R^2^ as an inadequate measure for nonlinear models in pharmacological and biochemical research: A Monte Carlo approach. BMC Pharmacol. 2010;10: 1–11. 20529254 10.1186/1471-2210-10-6 PMC2892436

[46] Neumann FJ, Schmid-Scho¨nbein H, Ohlenbusch H. Temperature-dependence of red cell aggregation. Pflu¨gers Archiv. 1987;408: 524–530. 10.1007/BF00585080 3601639

[47] Bessonov N, Sequeira A, Simakov S, Vassilevskii Yu, Volpert V. Methods of Blood Flow Modelling. Math Model Nat. 2016;11: 1–25. 10.1051/mmnp/201611101

[48] Kim S, Popel AS, Intaglietta M, Johnson PC. Aggregate formation of erythrocytes in postcapillary venules. Am J Physiol Heart Circ Physiol. 2005;288: H584–H590. 10.1152/ajpheart.00690.2004 15458951

[49] Kim S, Zhen J, Popel AS, Intaglietta M, Johnson PC. Contributions of collision rate and collision efficiency to erythrocyte aggregation in postcapillary venules at low flow rates. Am J Physiol Heart Circ Physiol. 2007;293: H1947–H1954. 10.1152/ajpheart.00764.2006 17616741

[50] Mehri, R. Red Blood Cell Aggregation Characterization: Quantification and modeling implications of red blood cell aggregation at low shear rates. PhD Thesis, University of Ottawa. 2016.

[51] Lipowsky H, USAMI S, Chien S. *In vivo* measurements of “apparent viscosity” and mcrovessel hematocrit in the mesentery of the cat. Microvasc Res. 1980;19: 297–319. 10.1016/0026-2862(80)90050-3 7382851

[52] Popel AS, Johnson PC. Microcirculation and Hemorheology. Annu Rev Fluid Mech. 2005;37: 43–69. 10.1146/annurev.fluid.37.042604.133933 21151769 PMC3000688

[53] Gross JF, Intaglietta M, Zweifach BW. Network model of pulsatile hemodynamics in the microcirculation of the rabbit omentum. Am J Physiol. 1974;226: 1117–1123. 10.1152/ajplegacy.1974.226.5.1117 4824864

